# Virulome analysis of *Escherichia coli* ST117 from bovine sources identifies similarities and differences with strains isolated from other food animals

**DOI:** 10.1371/journal.pone.0296514

**Published:** 2024-01-04

**Authors:** Bradd J. Haley, Serajus Salaheen, Seon Woo Kim, Jo Ann Van Kessel

**Affiliations:** Environmental Microbial and Food Safety Laboratory, Agricultural Research Service, United States Department of Agriculture, Beltsville, MD, United States of America; Cornell University, UNITED STATES

## Abstract

*Escherichia coli* ST117 is a pandemic extraintestinal pathogenic *E*. *coli* (ExPEC) causing significant morbidity globally. Poultry are a known reservoir of this pathogen, but the characteristics of ST117 strains from other animal sources have not been adequately investigated. Here we characterize the genomes of 36 ST117 strains recovered primarily from preweaned dairy calves, but also from older postweaned calves and lactating cows, in the context of other bovine-associated strains and strains from poultry, swine, and humans. Results of this study demonstrate that bovine-associated ST117 genomes encode virulence factors (VFs) known to be involved in extraintestinal infections, but also occasionally encode the Shiga toxin, a virulence factor (VF) involved in severe gastrointestinal infections and more frequently identified in *E*. *coli* from ruminants than other animals. Bovine-associated ST117 genomes were also more likely to encode *afa-VIII* (adhesins), *pap* (P-fimbriae), *cdt* (cytolethal distending toxin), and *stx* (Shiga toxins) than were poultry and swine-associated genomes. All of the ST117 genomes were grouped into seven virulence clusters, with bovine-associated genomes grouping into Clusters 1, 2, 4, 5, but not 3, 6, or 7. Major differences in the presence of virulence factors between clusters were observed as well. Antimicrobial resistance genes were detected in 112 of 122 (91%) bovine-associated genomes, with 103 of these being multidrug-resistant (MDR). Inclusion of genomes that differed from ST117 by one multi-locus sequence type (MLST) allele identified 31 STs, four of these among the bovine-associated genomes. These non-ST117 genomes clustered with the ST117 genomes suggesting that they may cause similar disease as ST117. Results of this study identify cattle as a reservoir of ST117 strains, some of which are highly similar to those isolated from other food animals and some of which have unique bovine-specific characteristics.

## Introduction

*Escherichia coli* is a diverse species of Gram-negative bacteria that are commensal members of the mammalian and avian gastrointestinal tracts and can be frequently isolated from the environment. Most *E*. *coli* strains are non-pathogenic, but the acquisition of virulence factors (VFs) can result in the emergence of pathogenic strains that can cause disease in humans and animals [1]. Currently, there are at least 11 pathovars (pathotypes) that result in various diseases caused by strains with specific combinations of VFs and that may be exacerbated by underlying conditions or age of the human host [2]. Treatment of these diseases typically includes antimicrobial therapy, except for infections caused by Shiga-toxigenic *E*. *coli* (STEC). However, the acquisition of antimicrobial resistance genes (ARGs) by these strains can make antimicrobial therapy ineffective or difficult to resolve.

Antimicrobial resistance remains a significant human and animal health concern causing increased morbidity and mortality from infections that are difficult to treat [3]. ARGs are frequently carried on mobile elements such as plasmids that can transfer between strains [4]. Dairy calves and cows and beef cattle are known reservoirs of antimicrobial-resistant bacteria, including MDR *E*. *coli* [5–8]. Dairy calves typically shed a greater ratio of resistant bacteria to susceptible bacteria, and the mechanisms for this are not yet elucidated [5, 9]. Previous research has suggested that diet is related to this difference in resistance carriage by animal age [10–12]. The iron content of milk is relatively low and this has been hypothesized to select for strains that encode accessory iron scavenging genes (siderophores) which are often colocated on plasmids carrying ARGs [11, 12].

*E*. *coli* ST117 is a globally distributed extraintestinal pathogenic *E*. *coli* (ExPEC) strain that primarily causes bladder infections [13, 14]. In the United States alone, such infections result in an estimated 7 million medical visits and $1.6 billion in medical expenses on an annual basis [15, 16]. Further, sepsis, which is frequently caused by ExPEC strains, has been estimated to cause more than 85,000 deaths annually [17]. Along with being a known ExPEC strain, ST117 is considered an avian pathogenic *E*. *coli* (APEC) as it causes colibacillosis which results in major economic losses for the broiler industry [18, 19]. Although ST117 has been well-characterized in human infections and poultry in recent years, strains from other animal sources, such as livestock, particularly dairy and beef cattle, are not well-characterized. The aim of this study was to investigate the genomic characteristics, particularly related to virulence and antimicrobial resistance, of ST117 strains from cows, and to compare these to genomes of the well-characterized poultry, swine, and human ST117 strains.

## Materials and methods

Genomes of 36 previously isolated *E*. *coli* ST117 strains recovered from dairy calves and cows in the United States were gathered from an in-house database of *E*. *coli* genomes (S1 File). Additionally, a subset of publicly-available ST117 genomes from bovine, poultry, swine, ovine, mustelid, and human sources were downloaded from the Enterobase database [20]. All available bovine, swine, ovine, mustelid, and human-isolated genomes were selected, but 1000 poultry-associated genomes were randomly selected to minimize computational resources while simultaneously analyzing a large number of genomes. Genomes that differed from ST117 genomes by one allele were simultaneously downloaded to identify any closely related non-ST117 genomes. To identify these genomes, the ST Query was set to 117, and the “Max Number MisMatches” in the Achtman 7 Gene MLST Query was set to 1.

All genomes were interrogated for the presence of virulence factors (VFs), antimicrobial resistance genes (ARGs), plasmid replicons, and serotype identification using the Abricate and STECFinder programs [21–23]. Clusters of similar virulence profiles (VPs) were identified with the Elbow Method [24] to determine the optimal number of clusters using the within-cluster sum of squares (WCSS) in the packages “cluster” and “pracma” in R. Differences in the virulome structures between clusters was determined by a PERMANOVA analysis in R. Differences in the proportions of VFs detected between clusters and between hosts (bovine versus poultry, and bovine versus swine) were determined using a Fisher’s exact test with the fisher.test command in the “stats” package in R. To account for false positive significant results (false significance at P < 0.05) a false discovery rate correction was applied to the Fisher’s exact test P-values with the p.adjust command in the “stats” package in R. Significance was considered at *P*_*adj*_ < 0.05.

## Results

### Diversity, virulence, and resistance among the bovine-associated strains

In total, the genomes of 1536 *E*. *coli* ST117 (including 36 in-house bovine-associated genomes) and closely related strains with defined source metadata (bovine, poultry, swine, mustelid, ovine, and human) were selected from the Enterobase database. Of these, 122 were from bovine sources, 257 were from humans, 1000 were from poultry, 152 were from swine, three were from mustelids, and two were from ovine sources (S2 File).

**Fig 1:**
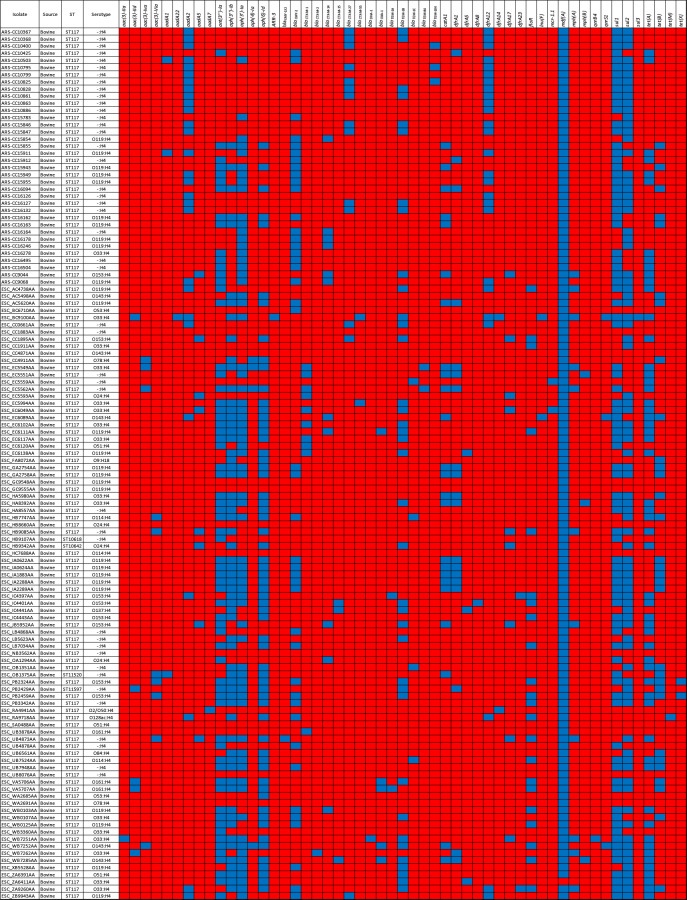
Sequence types (ST), serotypes, and presence/absence heatmap of antimicrobial resistance genes (ARGs) identified in bovine-associated ST117 genomes. Blue = present. Red = absent.

Of the 122 bovine-associated genomes, four were non-ST117 strains that differed from ST117 by one MLST allele (ST11597, ST11520, ST10642, and ST10618) (Fig 1) (S2 File). There were 15 different serotypes identified among the bovine isolates with O119:H4 being the most frequently detected (22% of all bovine isolates), followed by O33:H4 (13%), and O153:H4 (13%). Based on the genomic information, the O-antigen could not be determined in 36% of the genomes, but all of these were determined to encode the H4 H-antigen (labeled as -:H4) (Fig 1) (S2 File).

Of the 122 bovine genomes, 112 encoded at least one ARG. In total, 764 ARGs were detected, including genes that confer resistance to aminoglycosides, ß-lactams, tetracyclines, sulfonamides, trimethoprim, phenicols, quinolone, MLS, colistin, and rifamycin, in decreasing order of abundance (Fig 1; S3 File). On average, the bovine-associated genomes encoded 7 ARGs with aminoglycoside resistance being identified in 108 genomes, followed by sulfonamide resistance (104 genomes), ß-lactam resistance (93 genomes), tetracycline resistance (79 genomes), trimethoprim resistance (60 genomes) and phenicol resistance (44 genomes). On average, bovine-associated genomes encoded ARGs conferring resistance to four classes of antibiotics.

**Fig 2:**
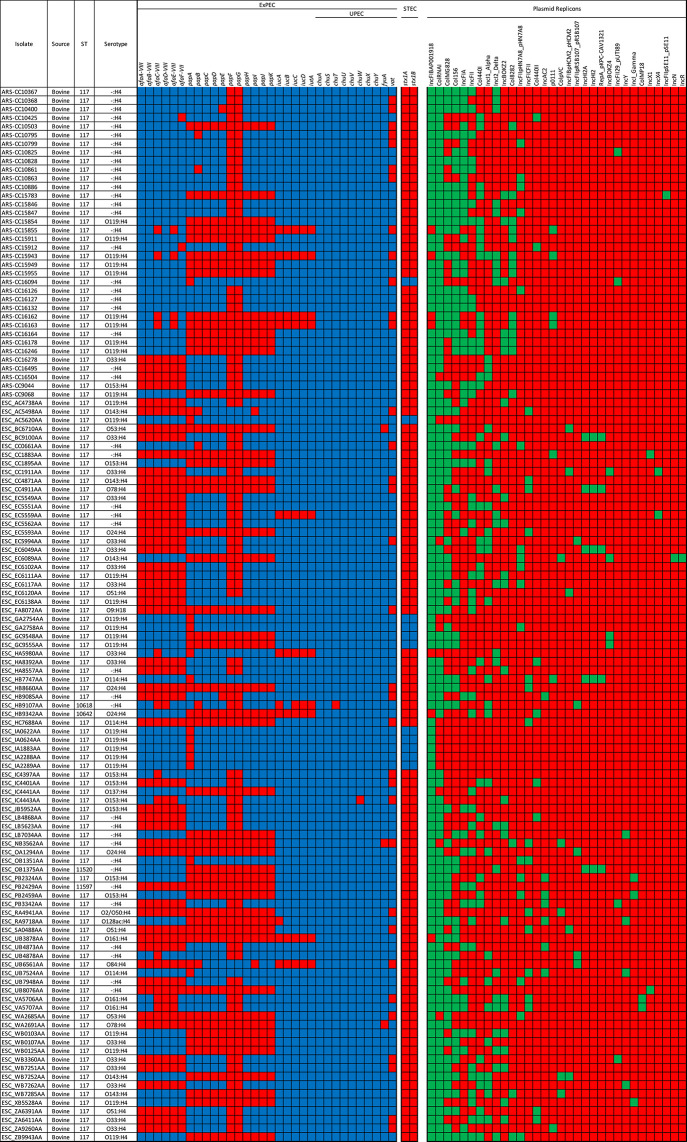
Presence/absence heatmap of major virulence factors (VFs) and plasmid replicons in the bovine-associated genomes. For VFs, blue = present and red = absent. For plasmid replicons, green = present and red = absent. ExPEC = extraintestinal pathogenic *E*. *coli*. UPEC = uropethogenic *E*. *coli*. STEC = shigatoxigenic *E*. *coli*.

A large-scale analysis of genes involved in virulence identified between 155 and 207 VFs in the 122 bovine-associated genomes (median = 184, mean = 182.2) (S4 File). The ST11597, ST11520, ST10642, and ST10618 genomes encoded 168, 193, 156, and 176 VFs, respectively. The presence/absence of VFs was variable across the bovine-associated strains, even for those VFs known to be integral or involved in the ExPEC infection processes (Fig 2). For instance, 57% of these genomes encoded at least one of the *afa* genes (*afa* binding genes), 59% encoded at least one *pap* gene (P fimbriae), and 92% encoded at least one *iucABCD-iutA* gene (aerobactin synthesis and receptor). The *foc* (F1C fimbriae), *sfa* (S fimbriae), *kpsMII* (group II capsule synthesis), *and dra* (adhesins) genes were not detected in any of the bovine-associated genomes.

Following the criteria of Johnson et al., [25], 102 genomes, including the ST11520, ST10642, and ST10618 genomes, were identified as potential ExPECs due to the presence of two of the following: *papA* and/or *papC*, *sfa* and/or *foc*, *afa* and/or *dra*, *kpsMII*, and *iutA* (Fig 2). Of the 20 genomes that did not meet these criteria, 19 were ST117 and one was ST11597. In total, 91 isolates were considered uropathogenic (UPEC) based on the presence of three or more of the following genes, *chuA*, *fyuA*, *vat*, *yfcV* [26]. None of the isolates encoded *yfcV*, including seven genomes that did not meet the ExPEC gene presence criteria and all the genomes with that had one allele difference from ST117 (ST11597, ST11520, ST10642, and ST10618). There were 24 genomes that met the ExPEC gene criteria but did not meet the UPEC criteria. All of these were lacking the *vat* gene.

The distribution of other non-ExPEC associated VFs indicated that some of the ST117 isolates were hybrid pathovars in that they encoded VFs that are integral in the pathogenesis of other pathovars (Fig 2). Of the 102 genomes with ExPEC genes, 11 encoded both *stx1A* and *stx2A* (Shiga toxin genes) of the STEC pathovar, and 10 of these genomes encoded the UPEC genes *vat*, *fyuA*, and *chuA* (ExPEC/STEC hybrid pathovar). None of the 11 genomes that encoded Shiga toxin genes, encoded the *eae* or *tir* genes.

There was a moderate dissimilarity between the virulomes of the bovine-associated and poultry-associated genomes as determined by an analysis of similarities test (ANOSIM R = 0.58, P < 0.001), with approximately 13% of the variance in virulome composition attributed to the distinction between the two groups (PERMANOVA, R^2^ = 0.13, F = 168.74, P < 0.001). Further, 58 VFs were significantly enriched in the bovine-associated genome group compared to the poultry-associated genome group (Table 1); enriched VFs included *pap* (P-fimbriae) genes, *afa-VIII* (adhesin) genes, *cdt-III* (cytolethal distending toxin) genes, and *stx* (Shiga toxin) genes (Fisher’s exact test, *P*_*adj*_ < 0.05). There were 40 VFs enriched in the poultry-associated genomes compared to the bovine-associated genomes, including *iro* (salmochelin) genes and the *sit* (iron transport genes). Similarly, moderate dissimilarities between the virulomes of bovine-associated and swine-associated genomes were identified, with approximately 20% of the variance in virulome composition attributed to the distinction between the two groups (ANOSIM R = 0.38, P < 0.001; PERMANOVA, R^2^ = 0.20, F = 70.161, P < 0.001). There were 54 VFs enriched in bovine isolates, including *pap*, *afa-VIII*, *cdt-III*, and *stx* genes, and 48 VFs enriched in swine-associated genomes, including *iro* and *sit* genes (Fisher’s exact test, *P*_*adj*_ < 0.05) (Table 2). Comparisons of the virulomes of both poultry-associated and swine-associated strains to bovine-associated strains indicate significant moderate differences between these groups as demonstrated by both ANOSIM R statistics. Based on the PERMANOVA R^2^ results, a higher percentage of the variation between the groups was explained for the comparions of swine-associated genomes to bovine-associated genomes (20%) than for the comparisons of poultry-associated genomes to bovine-associated genomes (13%). However, a comparison of the F-statistics indicates that the statistical significance of the poultry-associated genomes to bovine-associated genomes comparison is higher than that of the swine-associated genomes to bovine-associated genomes comparison.

**Table 1:** Virulence factors (VFs) enriched in bovine-associated genomes or poultry-associated genomes when the two groups are compared with each other. Results are from a Fisher’s exact test followed by a p-value correction for multiple comparisons. P_*adj*_ < 0.05 indicates a significant difference in the abundance of VFs when bovine-associated genomes and poultry-associated genomes are compared.

Virulence Factor (VF)	*p*_*adj*_	Enriched in Bovine/Poultry	Differences in proportion of genomes (|bovine-poultry|)
*espP*	2.01 × 10^−86^	Bovine	0.64
*afaA-VIII*	4.26 × 10^−73^	Bovine	0.56
*afaB-VIII*	4.26 × 10^−73^	Bovine	0.56
*afaF-VII*	1.8 × 10^−72^	Bovine	0.55
*clpB*	6.56 × 10^−69^	Bovine	0.52
*clpE*	6.56 × 10^−69^	Bovine	0.52
*clpG*	6.56 × 10^−69^	Bovine	0.52
*afaD-VIII*	1.75 × 10^−68^	Bovine	0.53
*faeJ*	1.75 × 10^−68^	Bovine	0.53
*afaE-VIII*	8.66 × 10^−65^	Bovine	0.51
*clpC*	1.24 × 10^−64^	Bovine	0.52
*clpF*	1.24 × 10^−64^	Bovine	0.52
*clpH*	1.24 × 10^−64^	Bovine	0.52
*clpL*	1.24 × 10^−64^	Bovine	0.52
*faeD*	1.24 × 10^−64^	Bovine	0.52
*afaC-VIII*	4.22 × 10^−60^	Bovine	0.47
*iroE*	2.25 × 10^−54^	Poultry	0.66
*iroD*	5.08 × 10^−53^	Poultry	0.66
*iroB*	7.59 × 10^−52^	Poultry	0.65
*iroC*	7.3 × 10^−51^	Poultry	0.65
*iroN*	3 × 10^−46^	Poultry	0.63
*focX*	5.2 × 10^−30^	Bovine	0.35
*int*	1.09 × 10^−22^	Bovine	0.47
*papA*	1.47 × 10^−21^	Bovine	0.38
*mchf*	1.47 × 10^−16^	Poultry	0.40
*ce1a*	1.99 × 10^−16^	Bovine	0.40
*fyuA*	4.49 × 10^−16^	Bovine	0.32
*ybtS*	4.49 × 10^−16^	Bovine	0.32
*ybtX*	4.49 × 10^−16^	Bovine	0.32
*irp2*	1.03 × 10^−15^	Bovine	0.33
*ybtU*	4.01 × 10^−15^	Bovine	0.32
*ybtA*	4.01 × 10^−15^	Bovine	0.31
*ybtE*	4.01 × 10^−15^	Bovine	0.31
*ybtQ*	4.01 × 10^−15^	Bovine	0.31
*ybtT*	4.01 × 10^−15^	Bovine	0.31
*cvac*	4.77 × 10^−14^	Poultry	0.34
*papI*	6.47 × 10^−14^	Bovine	0.35
*ybtP*	4.27 × 10^−13^	Bovine	0.30
*papD*	2.93 × 10^−12^	Bovine	0.33
*papJ*	2.93 × 10^−12^	Bovine	0.33
*papC*	6.14 × 10^−12^	Bovine	0.33
*papB*	6.33 × 10^−12^	Bovine	0.32
*papK*	1.81 × 10^−11^	Bovine	0.33
*papH*	2.69 × 10^−11^	Bovine	0.32
*cdt-IIIB*	9.57 × 10^−11^	Bovine	0.09
*cdt-IIIC*	9.57 × 10^−11^	Bovine	0.09
*stx1A*	9.57 × 10^−11^	Bovine	0.09
*stx1B*	9.57 × 10^−11^	Bovine	0.09
*papE*	1.55 × 10^−10^	Bovine	0.31
*hma*	4.33 × 10^−10^	Poultry	0.23
*cdt-IIIA*	8.77 × 10^−10^	Bovine	0.08
*cnf2*	8.77 × 10^−10^	Bovine	0.08
*traJ*	7.60 × 10^−9^	Bovine	0.26
*hlyC*	8.44 × 10^−9^	Bovine	0.08
*ecpB*	1.03 × 10^−8^	Poultry	0.09
*ecpA*	7.63 × 10^−8^	Poultry	0.09
*ecpD*	2.07 × 10^−7^	Poultry	0.09
*ecpC*	2.14 × 10^−7^	Poultry	0.09
*ecpE*	5.41 × 10^−7^	Poultry	0.08
*cei*	2.72 × 10^−6^	Bovine	0.06
pECS88_0104	6.60 × 10^−6^	Poultry	0.15
*epeA*	6.61 × 10^−6^	Bovine	0.05
*irp1*	8.3 × 10^−6^	Poultry	0.23
*sitB*	1.85 × 10^−5^	Poultry	0.17
*aatA*	3.48 × 10^−5^	Poultry	0.18
*sitC*	4.68 × 10^−5^	Poultry	0.12
*sitA*	1.86 × 10^−4^	Poultry	0.11
*leoa*	3.09 × 10^−4^	Bovine	0.05
*csa*	3.42 × 10^−4^	Poultry	0.10
*agn43*	4.25 × 10^−4^	Bovine	0.17
*flgE*	5.21 × 10^−4^	Poultry	0.03
*ompt*	0.00105	Poultry	0.13
*cma*	0.00155	Poultry	0.13
*cdiB*	0.00194	Bovine	0.14
*espL1*	0.00222	Poultry	0.04
*cda*	0.00223	Bovine	0.03
*mchb*	0.00455	Poultry	0.12
*flhD*	0.0047	Poultry	0.04
*cia*	0.00639	Bovine	0.05
*pixC*	0.00642	Poultry	0.10
*ccdb*	0.00767	Bovine	0.14
avian*-hlyE*	0.00955	Poultry	0.09
*papX*	0.0105	Bovine	0.04
*papF*	0.0105	Poultry	0.12
*aec7*	0.0114	Poultry	0.04
*papG*	0.0141	Poultry	0.12
*iss2*	0.0149	Poultry	0.04
*pixH*	0.0171	Poultry	0.08
*tia*	0.0227	Bovine	0.11
*pixF*	0.0235	Poultry	0.08
*pixB*	0.0235	Poultry	0.08
*wbdi*	0.0314	Poultry	0.05
*pixJ*	0.0321	Poultry	0.08
*ecpR*	0.0321	Poultry	0.04
*c3610*	0.0337	Poultry	0.11
*sfpC*	0.0338	Bovine	0.02
*cfa*	0.047	Bovine	0.03

**Table 2:** Virulence factors (VFs) enriched in bovine-associated genomes or swine-associated genomes when the two groups are compared with each other. Results are from a Fisher’s exact test followed by a p-value correction for multiple comparisons. P_*adj*_ < 0.05 indicates a significant difference in the abundance of VFs when bovine-associated genomes and swine-associated genomes are compared.

Virulence Factor (VF)	*p*_*adj*_	Enriched in Bovine/Swine	Differences in proportion of genomes (|bovine-swine|)
*espP*	5.06 × 10^−30^	Bovine	0.62
*afaA-VIII*	1.93 × 10^−26^	Bovine	0.55
*afaB-VIII*	1.93 × 10^−26^	Bovine	0.55
*faeJ*	9.92 × 10^−26^	Bovine	0.53
*afaF-VII*	1.54 × 10^−25^	Bovine	0.54
*clpB*	1.76 × 10^−25^	Bovine	0.52
*clpE*	1.76 × 10^−25^	Bovine	0.52
*clpG*	1.76 × 10^−25^	Bovine	0.52
*katp*	2.21 × 10^−25^	Swine	0.54
*afaD-VIII*	9.71 × 10^−25^	Bovine	0.52
*clpC*	2.25 × 10^−24^	Bovine	0.51
*clpF*	2.25 × 10^−24^	Bovine	0.51
*clpH*	2.25 × 10^−24^	Bovine	0.51
*clpL*	2.25 × 10^−24^	Bovine	0.51
*faeD*	2.25 × 10^−24^	Bovine	0.51
*afaE-VIII*	2.46 × 10^−23^	Bovine	0.50
*afaC-VIII*	2.73 × 10^−21^	Bovine	0.46
*iroB*	3.26 × 10^−15^	Swine	0.49
*iroC*	3.26 × 10^−15^	Swine	0.49
*tia*	8.33 × 10^−15^	Bovine	0.47
*iroD*	8.61 × 10^−15^	Swine	0.48
*iroE*	8.61 × 10^−15^	Swine	0.48
*iroN*	2.14 × 10^−14^	Swine	0.48
*fliD*	4.04 × 10^−14^	Swine	0.35
*focX*	1.17 × 10^−13^	Bovine	0.35
*ce1a*	3.94 × 10^−11^	Bovine	0.42
*ybtX*	1.96 × 10^−10^	Bovine	0.30
*irp2*	2.96 × 10^−10^	Bovine	0.30
*fyuA*	3.76 × 10^−10^	Bovine	0.29
*ybtS*	3.76 × 10^−10^	Bovine	0.29
*ybtE*	1.03 × 10^−9^	Bovine	0.29
*ybtQ*	1.03 × 10^−9^	Bovine	0.29
*ybtT*	1.03 × 10^−9^	Bovine	0.29
*ybtU*	1.03 × 10^−9^	Bovine	0.29
*mcja*	1.35 × 10^−9^	Swine	0.23
*ybtA*	1.99 × 10^−9^	Bovine	0.28
*papA*	5.87 × 10^−9^	Bovine	0.34
*mchf*	3.39 × 10^−8^	Swine	0.34
*ybtP*	3.70 × 10^−8^	Bovine	0.27
*ireA*	1.33 × 10^−7^	Bovine	0.27
*sitB*	2.45 × 10^−7^	Swine	0.24
*cma*	1.16 × 10^−6^	Swine	0.26
*cdiA*	4.71 × 10^−6^	Swine	0.25
*cvac*	7.82 × 10^−6^	Swine	0.23
*ccdb*	8.52 × 10^−6^	Bovine	0.27
*sitC*	2.33 × 10^−5^	Swine	0.15
*papI*	4.56 × 10^−5^	Bovine	0.27
*int*	5.22 × 10^−5^	Bovine	0.27
*irp1*	8.92 × 10^−5^	Swine	0.26
*csa*	1.87 × 10^−4^	Swine	0.14
*aatA*	2.69 × 10^−4^	Bovine	0.25
*papB*	3.60 × 10^−4^	Bovine	0.24
*papC*	3.85 × 10^−4^	Bovine	0.24
*papD*	3.85 × 10^−4^	Bovine	0.24
*papH*	3.85 × 10^−4^	Bovine	0.24
*papJ*	3.85 × 10^−4^	Bovine	0.24
*papK*	3.85 × 10^−4^	Bovine	0.24
*ehaA*	4.12 × 10^−4^	Bovine	0.12
*papF*	0.000509	Swine	0.20
*agn43*	0.000792	Bovine	0.21
*papE*	0.000968	Bovine	0.23
*papG*	0.00124	Swine	0.18
*sitA*	0.00256	Swine	0.12
*cdt-IIIB*	0.00538	Bovine	0.08
*cdt-IIIC*	0.00538	Bovine	0.08
*stx1A*	0.00538	Bovine	0.08
*stx1B*	0.00538	Bovine	0.08
*ecpB*	0.0061	Swine	0.09
*ecpD*	0.0061	Swine	0.09
*papX*	0.00731	Swine	0.12
*cdt-IIIA*	0.01	Bovine	0.08
*cnf2*	0.01	Bovine	0.08
*hlyC*	0.01	Bovine	0.08
*sitD*	0.0102	Swine	0.11
*ecpC*	0.0111	Swine	0.08
*ecpE*	0.0111	Swine	0.08
*cah*	0.0151	Bovine	0.16
avian*-hlyE*	0.0209	Swine	0.10
*epeA*	0.0223	Bovine	0.05
*flhD*	0.0223	Swine	0.05
*mccb*	0.0287	Swine	0.05
*papa_12*	0.0287	Swine	0.05
*ecpA*	0.0339	Swine	0.07
*cka*	0.0429	Swine	0.10
*clbA*	0.0446	Swine	0.05
*clbB*	0.0446	Swine	0.05
*clbC*	0.0446	Swine	0.05
*clbD*	0.0446	Swine	0.05
*clbE*	0.0446	Swine	0.05
*clbF*	0.0446	Swine	0.05
*clbG*	0.0446	Swine	0.05
*clbH*	0.0446	Swine	0.05
*clbI*	0.0446	Swine	0.05
*clbJ*	0.0446	Swine	0.05
*clbL*	0.0446	Swine	0.05
*clbM*	0.0446	Swine	0.05
*clbN*	0.0446	Swine	0.05
*clbO*	0.0446	Swine	0.05
*clbP*	0.0446	Swine	0.05
*clbQ*	0.0446	Swine	0.05
*clbR*	0.0446	Swine	0.05

There were 32 different plasmid replicon types detected among the bovine-associated genomes (Fig 2). The most frequently detected replicons were IncFIB(AP001918), ColRNAI, and Col(MG828), which were detected in 115, 107, and 67 genomes, respectively. Plasmid replicons were detected in all bovine-associated genomes with the range of detected replicons being 1 to 10 per genome (mean = 5.4, median = 5).

### Virulence profile and cluster identification among all genomes

**Fig 3:**
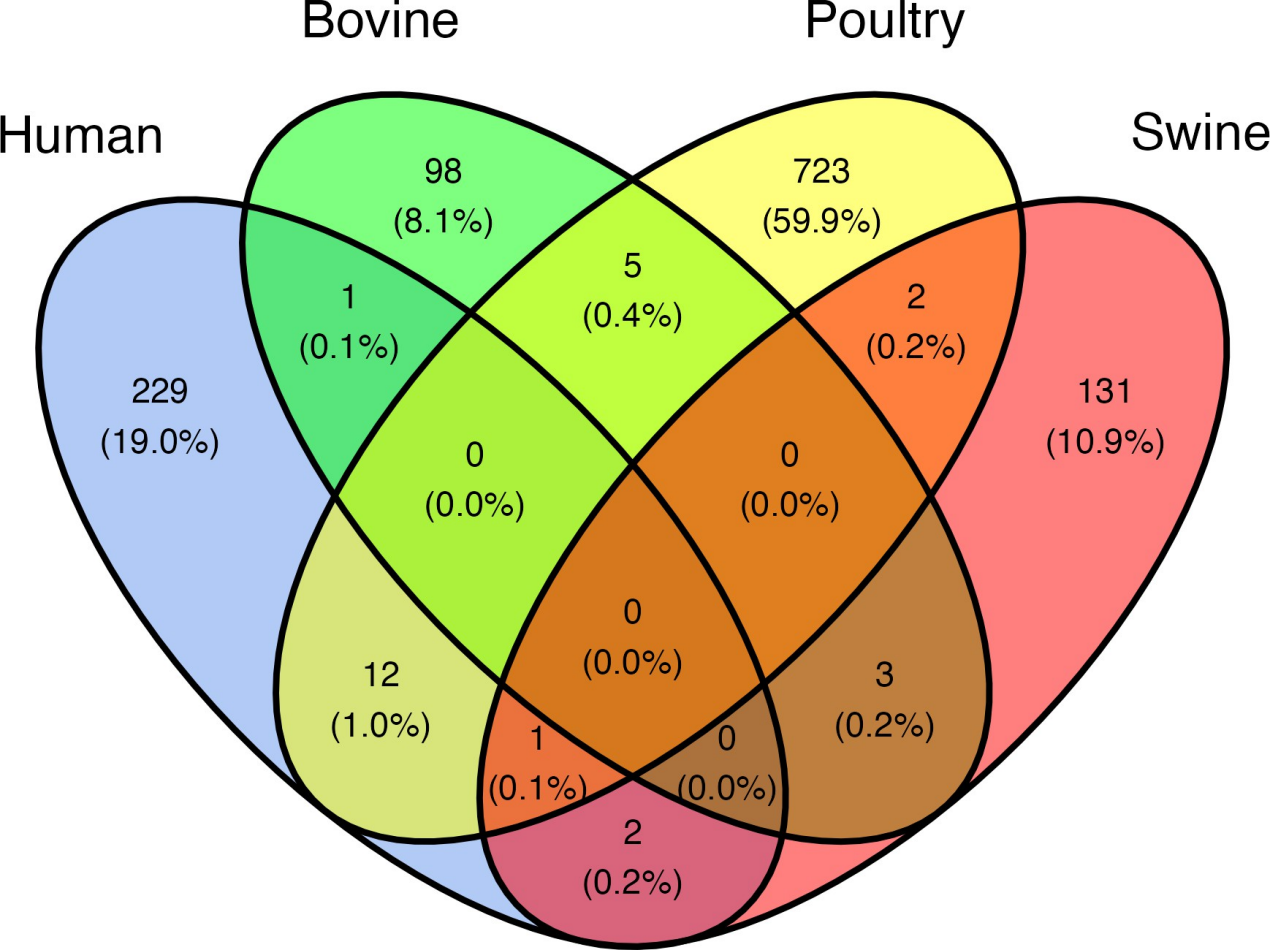
Venn diagram showing the number of virulence profiles (VP) identified among the genomes from each source and those identified in genomes from multiple sources. The number in the parentheses indicates the percentage of total VP.

There were 1204 unique virulence profiles (VPs), labeled as VP1 through VP1204, identified in the genomes (S5 File). There were 150 VPs that were identified in more than one genome, and 22 that were identified in more than five genomes. The most frequently identified VPs were VP334, VP611, and VP821 which were identified in 22, 20, and 17 genomes, respectively. In total, there were 98 VPs among the bovine-associated genomes that were not identified in the genomes from any other source animal, five VPs were shared with poultry-associated genomes, three VPs were shared with swine-associated genomes, and one VP was shared with human-isolated genomes (VP178) (Fig 3). The STEC strains were assigned to VP1-VP16, VP248-VP250, and VP347.

Based on the elbow method, the optimal number of clusters of similar VPs was 7 to 9 (there are multiple closely related VPs within a cluster). To minimize the number of clusters with very few genomes, seven clusters (labeled as Cluster 1 through Cluster 7) were selected for downstream analyses (Table 3). Clusters 2, 4, and 5 encompassed the most genomes with 636, 394, and 385, respectively. Clusters 1, 6, 7, and 3 were comprised of 56, 54, 7, and 4 genomes, respectively. Bovine-associated isolates belonged to Clusters 1, 2, 4, and 5; with Cluster 5 containing the most bovine-associated isolates (n = 64) followed by 1 (n = 40), 2 (n = 15), and 4 (n = 3). Cluster 4 was the only cluster comprised of isolates from all host animal sources. STEC strains were assigned to Clusters 1, 2, 3, and 4.

**Table 3:** Composition of the virulence profile (VP) clusters by source (rows) and cluster assignments of the genomes (columns). The first number represents the number of genomes from each source assigned to that cluster, the first number in the parentheses represent the percentage of genomes from that cluster isolated from each source, and the second number in the parentheses represents the percentage of total isolates from that source that are assigned to each cluster.

Source	Cluster 1	Cluster 2	Cluster 3	Cluster 4	Cluster 5	Cluster 6	Cluster 7
Bovine	40 (71%, 16%)	15 (2%, 12%)	0	3 (0.7%, 2.4%)	64 (14%, 52%)	0	0
Human	14 (25%, 5%)	141 (22%, 52%)	3 (75%, 1%)	33 (8.3%, 12%)	66 (14%, 24%)	14 (25%, 5%)	0
Mustelid	0	1 (0.1%, 25%)	0	1 (0.2%, 25%)	1 (0.2%, 25%)	0	0
Ovine	0	0	0	2 (0.5%, 100%)	0	0	0
Poultry	0	419 (65%, 40%)	1 (25%, 25%)	322 (81%, 31%)	258 (57%, 25%)	24 (44%, 2%)	0
Swine	2 (4%, 1%)	60 (9%, 34%)	0	33 (8%, 18%)	57 (12%, 32%)	16 (29%, 29%)	7 (100%, 4%)
Total	56	636	4	394	446	54	7

**Fig 4:**
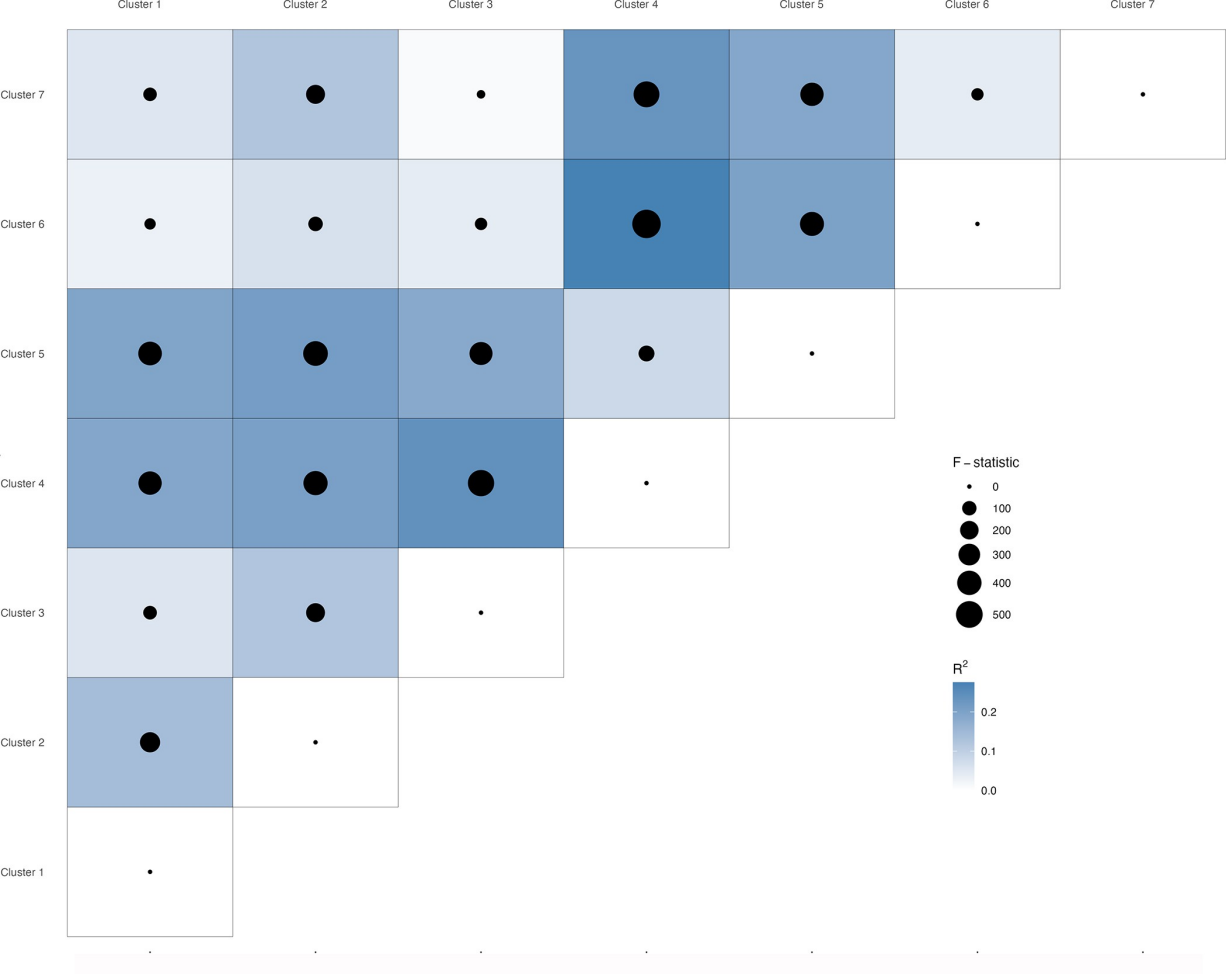
Differences in the virulome structures of the clusters as determined by a PERMANOVA analysis. The darkness of the blue squares is proportional to the R^2^ values. The size of the black circle is proportional to the F-statistic.

**Fig 5:**
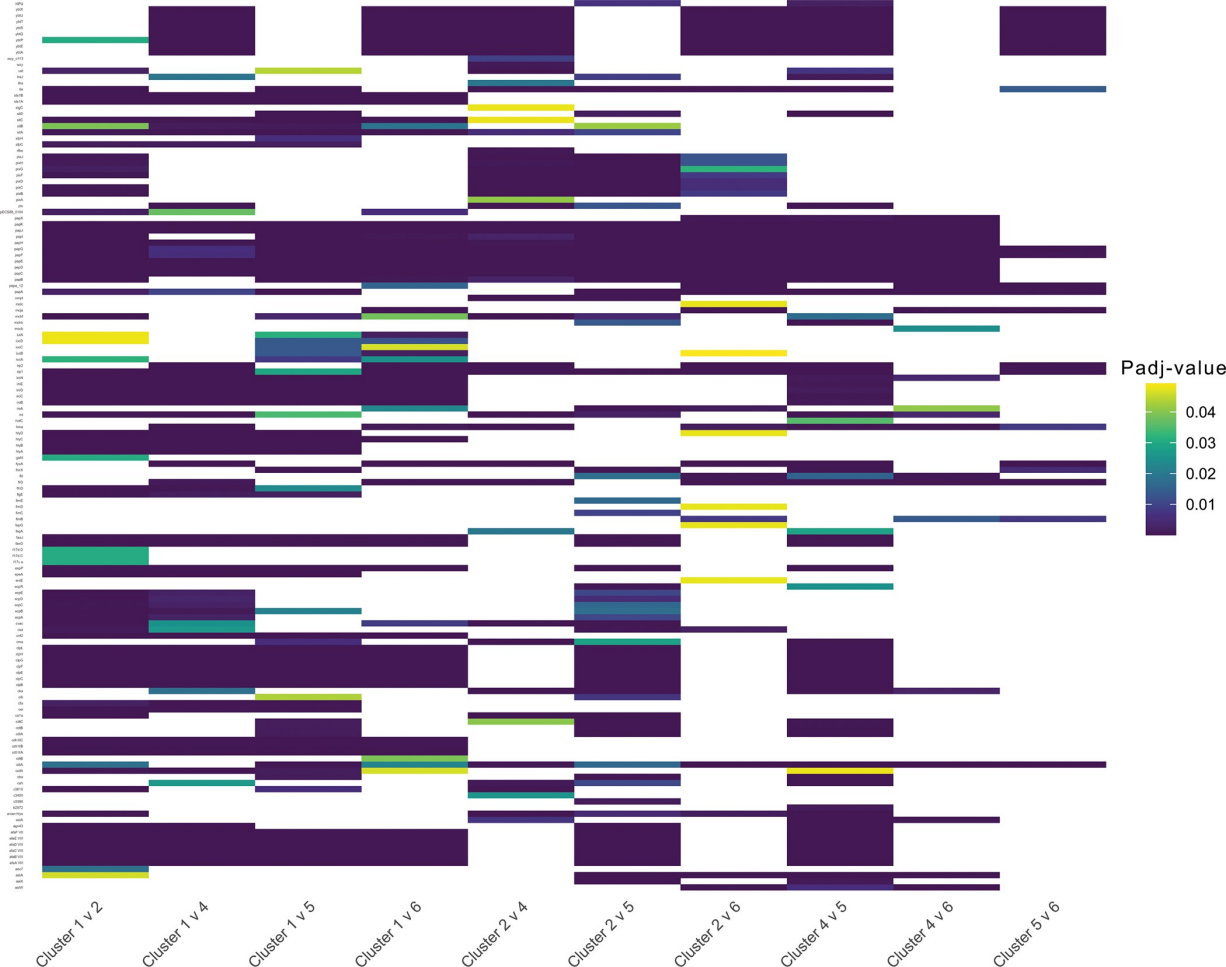
Heatmap showing the virulence factors (VFs) that are enriched in one cluster compared to another cluster. Yellow to purple scale = decreasing P_*adj*_-values from 0.05. White = P_*adj*_-value > 0.05.

There were significant differences in virulome structure between these clusters (Fig 4), with Clusters 4 and 5 having the most apparent differences with other clusters, but fewer differences with each other (PERMANOVA, R^2^ = 0.07 to 0.27, F-statistic = 132 to 582, P-value < 0.05). When considering Clusters 4 and 5, 7% to 27% of the total variance in VF presence and absence patterns was attributed to the grouping variable with other clusters, with varying degrees of significance. A differential enrichment of some VFs was also observed between the clusters. Of 380 VFs, 145 were differentially abundant in at least one cluster, eight were differentially abundant in two clusters, 29 were differentially abundant in 3 clusters, and 27 were differentially abundant in four clusters. Of all of the cluster comparisons, Clusters 1 and 2 had the most differentially enriched VFs between them, followed by Clusters 1 and 4, Clusters 4 and 5, Cluster 1 and 5, and Clusters 2 and 5 (Fig 5, S6 File).

## Discussion

*E*. *coli* ST117 is an APEC/ExPEC lineage and a major cause of human morbidity worldwide. Poultry is considered a major reservoir of ST117 [13, 27, 28]; however, strains from other sources, particularly bovine sources, have not been fully investigated. Recent studies have demonstrated that ST117 is isolated from these animals, particularly young dairy and veal calves, suggesting that these animals may be a potential reservoir of *E*. *coli* ST117 [11, 29, 30]. However, many fewer *E*. *coli* ST117 strains have been isolated from bovine sources compared with poultry sources so cattle may serve as a minor reservoir of these strains compared to poultry. The prevalence of ST117 in bovine sources in the United States has not been evaluated.

Most of the genomes in this study, regardless of source, encoded ARGs conferring resistance to multiple classes of antibiotics, and can be considered multidrug-resistant (MDR). The majority of the bovine-associated genomes encoded aminoglycoside, ß-lactam, tetracycline, and sulfonamide resistance genes. Antimicrobial administration data was not available for the animals from which these isolates were recovered, so associations between treatment and ARG presence in these strains could not be assessed. ST117 strains are frequently antimicrobial-resistant and frequently MDR, suggesting that mechanisms involved in the persistence of the MDR genotype may also be beneficial to this sequence type, or that the maintenance of ARGs does not confer a disadvantage on resistant ST117 strains compared with susceptible ST117 strains.

Multidrug-resistant *E*. *coli* ST117 can be found in the bovine calf gut, which is an iron-poor environment because of the low iron levels in colostrum and milk [11, 12, 29]. Previous research has identified a positive association between the presence of iron-scavenging genes and the MDR genotype in *E*. *coli* collected from young calves [11, 12]. In this study, 81% of the bovine-associated genomes encoded *iucABCD-iutA*, 97% encoded *fyuA*, 99% encoded *chuA*, and 71% encoded *sitABCD*, all of which are involved in iron transport from the extracellular environment into the *E*. *coli* cell. Genomes of isolates from our collection (“ARS-CC” prefix) were predominantly collected from preweaned calves. A similar trend was observed in ST69 isolates from an earlier study, in which the majority of isolates encoded a *sitABCD* iron transport operon and were predominantly isolated from preweaned calves rather than evenly recovered from preweaned and postweaned calves [31]. Dairy calves are often born anemic and for the first eight weeks of life are predominantly fed a milk/milk-replacer diet, which is low in iron compared to the forages of older post-weaned animals [32]. We have previously hypothesized that this low-iron environment selects for strains that encode accessory siderophores, such as those strains with *iucABCD-iutA* and *sitABCD* [11, 12]. These two iron-scavenging operons are frequently located on an IncFIB plasmid that can simultaneously encodes ARGs [33, 34]. The seven bovine-associated genomes that do not encode an IncFIB plasmid do not encode these two scavenging systems. The high prevalence of the IncFIB plasmid encoding these scavenging systems in ST117 strains and other ExPEC strains may be a factor in the high prevalence of these strains in dairy calves. Further work is needed to evaluate this *in vivo*.

Variation in the virulence profiles was observed among the genomes and these variants grouped into clusters of similar profiles. The noticeable differences are clearly apparent with the frequency of certain VFs being higher in some clusters than others. This is in part due to the mobile nature of some of these VFs which can frequently be encoded in plasmids and genomic islands. These data suggest that there might be some differences in the virulence potentials of these clusters, but the differences would need to be evaluated *in vivo*. There was considerable overlap of isolates from different sources within the clusters suggesting that some similar strains may circulate between different hosts. For instance, poultry-associated isolates were recovered from four of seven virulome clusters (Clusters 2, 4, 5, and 6), and bovine-associated isolates were recovered from three of the same clusters (Clusters 2, 4, and 5). However, bovine-associated isolates were the major constituent (71% of isolates) of Cluster 1, which also included human isolates (25%), two swine isolates, and no poultry isolates.

Moderate variations in virulome structures were observed when bovine-associated genomes were compared to poultry and swine-associated genomes suggesting that host species may be involved in the selection of some VFs, many of which are mobile. Interestingly the bovine-associated isolates encoded some VFs that were identified less frequently in non-bovine-associated isolates. Shiga toxin genes (*stx*) were only identified in bovine-associated isolates and human-recovered isolates, although the health statuses of the latter were not available. While they appear to have no impact on cattle, Shiga toxins can cause severe illness, including hemolytic uremic syndrome (HUS) in approximately 5–10% of people infected with STECs and is sometimes fatal. ExPEC strains typically do not cause infections of the human gut leading to illness. However, the presence of *stx* indicates that the ST117 hybrid ExPEC/STEC strains may be able to cause both gastrointestinal infections and extraintestinal infections. Cattle are the primary reservoir of STEC [35, 36], yet this animal’s carriage of hybrid ExPEC/STEC strains has not been fully investigated and further work should be conducted to better understand the prevalence of these strains in dairy and beef animals, their public health significance, as well as their potential pathogenesis in humans.

Results of this study demonstrate that cows and calves are potential sources of *E*. *coli* ST117 and closely related non-ST117 strains, although poultry is most likely the major food animal reservoir of these strains. It appears that multiple food animals may be potential reservoirs of similar and potentially virulent strains. Our work further suggests that preweaned calves may be a primary bovine reservoir of ST117, ST69, and other STs that are frequent carriers of iron-scavenging genes, many of which are known to cause extraintestinal infections in humans. The presence of Shiga toxin genes in a small portion of the bovine-associated *E*. *coli* ST117 strains suggests these animals are potential sources of ExPEC/STEC hybrid strains. The public health significance of such strains should be more deeply evaluated. Infections cause by ExPEC strains can be difficult to treat due the high prevalence of resistance among these strains. Associations between iron-scavenging genes and ARGs in the bovine calf gut should be further evaluated in an effort to mitigate carriage of MDR ST117 and other virulent MDR *E*. *coli* strains by these animals.

## Supporting information

S1 File NCBI accession numbers for genomes sequenced for this study. (XLSX)

S2 File Strain ID, source, sequence type (ST), and serotype of strains utilized in the study. (XLSX)

S3 File Antimicrobial resistance genes (ARGs) of strains utilized in the study. (XLSX)

S4 File Virulence factors (VFs) of strains utilized in the study. (XLSX)

S5 File Virulence profiles (VPs) and clusters of strains utilized in the study. (XLSX)

S6 File Percentage of strains within a each cluster encoding each virulence factor. (XLSX)

## References

[1] Kaper JB, Nataro JP, Mobley HL. Pathogenic *Escherichia coli*. Nat Rev Microbiol. 2004 Feb;2(2):123–40. doi: 10.1038/nrmicro818 15040260

[2] Geurtsen J, de Been M, Weerdenburg E, Zomer A, McNally A, Poolman J. Genomics and pathotypes of the many faces of *Escherichia coli*. FEMS Microbiol Rev. 2022;46(6):fuac031. doi: 10.1093/femsre/fuac031 35749579 PMC9629502

[3] Prestinaci F, Pezzotti P, Pantosti A. Antimicrobial resistance: a global multifaceted phenomenon. Pathog Glob Health. 2015;109(7):309–18. doi: 10.1179/2047773215Y.0000000030 26343252 PMC4768623

[4] Oladeinde A, Cook K, Lakin SM, Woyda R, Abdo Z, Looft T, et al. Horizontal gene transfer and acquired antibiotic resistance in *Salmonella enterica* Sserovar Heidelberg following *in vitro* incubation in broiler ceca. Appl Environ Microbiol. 2019;85(22):e01903–19. doi: 10.1128/AEM.01903-19 31471306 PMC6821953

[5] Cao H, Pradhan AK, Karns JS, Hovingh E, Wolfgang DR, Vinyard BT, et al. Age-associated distribution of antimicrobial-resistant *Salmonella enterica* and *Escherichia coli* Iisolated from dairy herds in Pennsylvania, 2013–2015. Foodborne Pathog Dis. 2019;16(1):60–67. doi: 10.1089/fpd.2018.2519 30597121

[6] Salaheen S, Cao H, Sonnier JL, Kim SW, Del Collo LP, Hovingh E, et al. Diversity of extended-spectrum cephalosporin-resistant *Escherichia coli* in feces from calves and cows on Pennsylvania dairy farms. Foodborne Pathog Dis. 2019;16(5):368–370. doi: 10.1089/fpd.2018.2579 30715902

[7] Schmidt JW, Vikram A, Arthur TM, Belk KE, Morley PS, Weinroth MD, et al. Antimicrobial resistance at two U.S. cull cow processing establishments. J Food Prot. 2020;83(12):2216–2228. doi: 10.4315/JFP-20-201 32730612

[8] Manishimwe R, Moncada PM, Bugarel M, Scott HM, Loneragan GH. Antibiotic resistance among *Escherichia coli* and *Salmonella* isolated from dairy cattle feces in Texas. PLoS One. 2021;16(5):e0242390. doi: 10.1371/journal.pone.0242390 33961628 PMC8104409

[9] Springer HR, Denagamage TN, Fenton GD, Haley BJ, Van Kessel JAS, Hovingh EP. Antimicrobial resistance in fecal *Escherichia coli* and *Salmonella enterica* from dairy calves: a systematic review. Foodborne Pathog Dis. 2019;16(1):23–34. doi: 10.1089/fpd.2018.2529 30481058

[10] Liu J, Taft DH, Maldonado-Gomez MX, Johnson D, Treiber ML, Lemay DG, et al. The fecal resistome of dairy cattle is associated with diet during nursing. Nat Commun. 2019;10(1):4406. doi: 10.1038/s41467-019-12111-x 31562300 PMC6765000

[11] Haley BJ, Kim SW, Salaheen S, Hovingh E, Van Kessel JAS. Virulome and genome analyses identify associations between antimicrobial resistance genes and virulence factors in highly drug-resistant *Escherichia coli* isolated from veal calves. PLoS One. 2022;17(3):e0265445. doi: 10.1371/journal.pone.0265445 35298535 PMC8929554

[12] Haley BJ, Kim SW, Salaheen S, Hovingh E, Van Kessel JS. Genome-wide analysis of *Escherichia coli* isolated from dairy animals identifies virulence factors and genes enriched in multidrug-resistant strains. Antibiotics. 2023;12(10):1559. doi: 10.3390/antibiotics12101559 37887260 PMC10604827

[13] Manges AR. *Escherichia coli* and urinary tract infections: the role of poultry-meat. Clin Microbiol Infect. 2016;22(2):122–129. doi: 10.1016/j.cmi.2015.11.010 26679924

[14] Yamaji R, Friedman CR, Rubin J, Suh J, Thys E, McDermott P, et al. A Population-based surveillance study of shared genotypes of *Escherichia coli* isolates from retail meat and suspected cases of urinary tract infections. mSphere. 2018;3(4):e00179–18. doi: 10.1128/mSphere.00179-18 30111626 PMC6094058

[15] Foxman B, Barlow R, D’Arcy H, Gillespie B, Sobel JD. Urinary tract infection: self-reported incidence and associated costs. Ann Epidemiol. 2000;10(8):509–15. doi: 10.1016/s1047-2797(00)00072-7 11118930

[16] Foxman B. Epidemiology of urinary tract infections: incidence, morbidity, and economic costs. Dis Mon. 2003;49(2):53–70. doi: 10.1067/mda.2003.7 12601337

[17] Poolman JT, Wacker M. Extraintestinal pathogenic *Escherichia coli*, a common human pathogen: challenges for vaccine development and progress in the field. J Infect Dis. 2016;213(1):6–13. doi: 10.1093/infdis/jiv429 26333944 PMC4676548

[18] Ghunaim H., Abu-Madi M.A., Kariyawasam S. Advances in vaccination against avian pathogenic *Escherichia coli* respiratory disease: Potentials and limitations. Vet. Microbiol. 2014;172:13–22. doi: 10.1016/j.vetmic.2014.04.019 24878325

[19] de Brito B.G., Gaziri L.C.J., Vidotto M.C. Virulence factors and clonal relationships among *Escherichia coli* Sstrains isolated from broiler chickens with cellulitis. Infect. Immun. 2003;71:4175. doi: 10.1128/IAI.71.7.4175–4177.2003 12819112 PMC162012

[20] Zhou Z, Alikhan NF, Mohamed K, the Agama Study Group, Achtman M. The EnteroBase user’s guide, with case studies on *Salmonella* transmissions, *Yersinia pestis* phylogeny and *Escherichia* core genomic diversity. Genome Res. 2020;30:138–152. doi: 10.1101/gr.251678.119 31809257 PMC6961584

[21] Seeman, 2020. https://github.com/tseemann/abricate. Last accessed on 07/09/2023.

[22] Zankari E, Hasman H, Cosentino S, Vestergaard M, Rasmussen S, Lund O, et al. Identification of acquired antimicrobial resistance genes. J Antimicrob Chemother. 2012;67(11):2640–4. doi: 10.1093/jac/dks261 22782487 PMC3468078

[23] Carattoli A, Zankari E, García-Fernández A, Voldby Larsen M, Lund O, Villa L, et al. *In silico* detection and typing of plasmids using PlasmidFinder and plasmid multilocus sequence typing. Antimicrob Agents Chemother. 2014;58(7):3895–903. doi: 10.1128/AAC.02412-14 24777092 PMC4068535

[24] Nies HW, Zakaria Z, Mohamad MS, Chan WH, Zaki N, Sinnott RO, et al. A review of computational methods for clustering genes with similar biological functions. Processes. 2019;7(9):550. doi: 10.3390/pr7090550

[25] Johnson JR, Murray AC, Gajewski A, et al. Isolation and molecular characterization of nalidixic acid–resistant extraintestinal pathogenic *Escherichia coli* from retail chicken products, Antimicrob Agents Chemother, 2003;47:2161–8. doi: 10.1128/AAC.47.7.2161–2168.2003 12821463 PMC161843

[26] Spurbeck RR, Dinh PC Jr, Walk ST, Stapleton AE, Hooton TM, Nolan LK, et al. *Escherichia coli* isolates that carry vat, fyuA, chuA, and yfcV efficiently colonize the urinary tract. Infect Immun. 2012;80(12):4115–22. doi: 10.1128/IAI.00752-12 22966046 PMC3497434

[27] Manges AR, Geum HM, Guo A, Edens TJ, Fibke CD, Pitout JDD. Global extraintestinal pathogenic *Escherichia coli* (ExPEC) lineages. Clin Microbiol Rev. 2019;32(3):e00135–18. doi: 10.1128/CMR.00135-18 31189557 PMC6589867

[28] Manges AR. *Escherichia coli* causing bloodstream and other extraintestinal infections: tracking the next pandemic. Lancet Infect Dis. 2019;19(12):1269–1270. doi: 10.1016/S1473-3099(19)30538-9 31653525

[29] Kim SW, Karns JS, Van Kessel JAS, Haley BJ. Genome sequences of five multidrug-resistant *Escherichia coli* sequence type 117 isolates recovered from dairy calves. Genome Announc. 2017;5(33):e00732–17. doi: 10.1128/genomeA.00732-17 28818889 PMC5604762

[30] Kim SW, Van Kessel JAS, Haley BJ. Genome sequences of antibiotic-resistant *Escherichia coli* isolated from veal calves in the USA. J Glob Antimicrob Resist. 2021;26:69–73. doi: 10.1016/j.jgar.2021.04.024 34052521

[31] Salaheen S, Kim SW, Springer HR, Hovingh EP, Van Kessel JAS, Haley BJ. Characterization of antimicrobial resistance genes and virulence factors in the genomes of *Escherichia coli* ST69 isolates from preweaned dairy calves and their phylogenetic relationship with poultry and human clinical strains. Microb Drug Resist. 2023; 29(6):249–255. doi: 10.1089/mdr.2022.0266 36961425

[32] Budny-Walczak A, Śpitalniak-Bajerska K, Szołtysik M, Pogoda-Sewerniak K, Kupczyński R. Effects of iron supplementation on metabolism in calves receiving whole milk. Animals (Basel). 2023;13(3):477. doi: 10.3390/ani13030477 36766366 PMC9913521

[33] Aljahdali NH, Khajanchi BK, Weston K, Deck J, Cox J, Singh R, et al. Genotypic and phenotypic characterization of incompatibility group FIB positive *Salmonella enterica* serovar Typhimurium isolates from food animal sources. Genes (Basel). 2020; 11(11):1307. doi: 10.3390/genes11111307 33158112 PMC7716204

[34] Abbott CN, Felix M, Foley SL, Khajanchi BK. Expression of genes located on the incompatibility group FIB plasmids at transcription and protein levels in iron-modified growth conditions. Front Microbiol. 2021;12:729275. doi: 10.3389/fmicb.2021.729275 34803945 PMC8602916

[35] Moxley RA. *Escherichia coli* 0157:H7: an update on intestinal colonization and virulence mechanisms. Anim Health Res Rev. 2004;5(1):15–33. doi: 10.1079/ahr200463 15460538

[36] Menge C. (2020). The role of *Escherichia coli* Shiga toxins in STEC colonization of cattle. Toxins, 12(9), 607 doi: 10.3390/toxins12090607 32967277 PMC7551371

